# Long-term risk of all-cause mortality and cardiovascular events in women with gestational diabetes mellitus: a systematic review and meta-analysis

**DOI:** 10.3389/fendo.2026.1646691

**Published:** 2026-02-25

**Authors:** Hong Zeng, Xiaoping Yin, Li Yin, Qi Chen, Fei Yang, Yuanjunzi Shi, Danqing Zhao, Heng Luo

**Affiliations:** 1 Department of Obstetrics and Gynecology, Affiliated Hospital of Guizhou Medical University, Guiyang, Guizhou, China; 2 State Key Laboratory of Discovery and Utilization of Functional Components in Traditional Chinese Medicine, Natural Products Research Center of Guizhou Province, Guizhou Medical University, Guiyang, China

**Keywords:** all - cause mortality, cardiovascular events, gestational diabetes mellitus, long - term risk, meta - analysis, systematic review

## Abstract

**Background:**

This meta-analysis investigates the long-term association between gestational diabetes mellitus (GDM) and the risks of all-cause mortality and specific cardiovascular events in women.

**Methods:**

We analyzed 9 high-quality cohort studies involving 4,191,840 women (age range: 24.5-34.6 years).

**Results:**

Compared to women without GDM, those with a history of GDM had significantly increased risks of: All-cause mortality (HR = 1.29, 95% CI 1.09-1.52),Acute heart failure (HR = 1.74, 95% CI 1.36-2.23), Myocardial infarction (HR = 1.63, 95% CI 1.38-1.91), Ischemic stroke (HR = 1.70, 95% CI 1.28-2.26). Heterogeneity was observed for all outcomes except myocardial infarction. Sensitivity analyses confirmed the robustness of the findings. The absolute incidence of all-cause mortality was also higher in the GDM group (3.2% vs. 2.5%). No significant publication bias was detected.

**Conclusion:**

GDM is significantly associated with elevated long-term risks of all-cause mortality and cardiovascular morbidity. These findings underscore the necessity for long-term monitoring and preventive strategies in this population, even after postpartum glucose normalization.

**Systematic review registration:**

https://www.crd.york.ac.uk/PROSPERO/, identifier CRD42025649099.

## Introduction

Gestational Diabetes Mellitus (GDM) refers to glucose intolerance first diagnosed during pregnancy, excluding diabetes that existed before pregnancy. Its typical onset period is the middle to late stages of pregnancy (24–28 weeks), and its core pathological features include increased insulin resistance, elevated blood glucose levels, and glucose metabolism disorders (1, 2). According to epidemiological data from the International Diabetes Federation (IDF) and the World Health Organization (WHO), the global prevalence of GDM ranges from 5% to 20%. However, due to differences in regions, ethnic groups, and diagnostic criteria, the prevalence varies significantly among different populations. For example, the prevalence of GDM in the Asian population is significantly higher than that in other ethnic groups due to factors such as genetic susceptibility (e.g., TCF7L2 gene polymorphism) and dietary structure (high carbohydrate intake) (2, 3). In recent years, with the global aging population, the rising obesity rate (especially abdominal obesity), and the popularity of a sedentary lifestyle, the prevalence of GDM has shown a continuous upward trend, making it one of the most common metabolic complications during pregnancy (4, 5).

The high - risk factors for GDM have been widely verified, including pre - pregnancy overweight/obesity (Body Mass Index (BMI) ≥ 25 kg/m²), insulin resistance, Polycystic Ovary Syndrome (PCOS), family history of diabetes, and pregnancy - related factors such as advanced maternal age (≥ 35 years), multiple pregnancies, and a previous history of GDM (4, 6). These factors exacerbate glucose metabolism abnormalities through a synergistic effect, further increasing the risk of GDM.

The short - term adverse effects of GDM on pregnancy outcomes are clear. For the mother, it can increase the risk of gestational hypertension, preeclampsia, polyhydramnios, and the rate of cesarean section. For the fetus and neonate, it can lead to macrosomia (birth weight ≥ 4000 g), preterm birth, neonatal hypoglycemia, respiratory distress syndrome, and neonatal jaundice (7, 8). In clinical practice, the management of GDM during pregnancy is based on lifestyle interventions (dietary control, regular exercise), supplemented by blood glucose monitoring, and insulin is used to control blood glucose if necessary (9). However, a growing number of studies have shown that the impact of GDM on women’s health is not limited to pregnancy. Even if blood glucose returns to normal after childbirth, women with GDM still face long - term risks of metabolic and cardiovascular health problems, including Type 2 Diabetes Mellitus (T2DM), metabolic syndrome (central obesity, hypertension, hyperglycemia, hyperlipidemia), and Cardiovascular Disease (CVD) (4, 10).

Previous cohort studies have initially explored the association between GDM and long - term health. For example, a prospective study by Tobias et al. (10) (including a cohort of American women) found that the risk of cardiovascular events in women with GDM was more than 30% higher than that in women without GDM 10–20 years after childbirth, and this association was independent of the occurrence of T2DM. A study by Wang et al. (11) further confirmed that the all - cause mortality rate of women with GDM was 29% higher than that of the control group, mainly due to cardiovascular diseases and diabetes - related complications. However, some studies [such as the UK Biobank study by Lee et al. (12)] did not find a significant association between GDM and long - term cardiovascular events. This difference may be due to variations in study design (e.g., follow - up duration, sample size), population characteristics (ethnicity, socioeconomic status), and control of confounding factors (e.g., BMI, postnatal lifestyle) (12, 13). In addition, most existing studies focus on a single outcome (e.g., only T2DM or myocardial infarction) and lack a comprehensive quantitative analysis of all - cause mortality and various cardiovascular events, making it difficult to fully reveal the long - term health impact of GDM (14, 15).

Based on the aforementioned research background of GDM, as a common metabolic complication of pregnancy, has attracted increasing attention regarding its association with women’s long-term health. Although some studies suggest that GDM may elevate the risks of cardiovascular disease and mortality, the existing evidence lacks systematic synthesis, particularly regarding the long-term risk assessment for specific types of cardiovascular events. Therefore, this systematic review and meta-analysis aims to synthesize current high-quality cohort studies to comprehensively quantify the long-term risks of all-cause mortality, acute heart failure, myocardial infarction, ischemic stroke, and other related outcomes in women with a history of GDM. By utilizing large-scale data and rigorous analytical methods, this study seeks to provide an evidence-based foundation for developing clinical strategies for long-term follow-up and cardiovascular disease prevention in this population.

## Methods

This systematic review and meta-analysis adhered to the Preferred Reporting Items for SystematicReviews and Meta-analyses (PRISMA) guidelines (see **Supplementary Material 2**, PRISMA Checklist) and was prospectively registered with PROSPERO (CRD42025649099).

### Literature search

A combination of MeSH terms and free words was used to search for relevant cohort studies in PubMed, Web of Science, Embase, and Cochrane Library databases up to February 15, 2025. The search terms included: “gestational diabetes mellitus”, “gestational diabetes”, “GDM” (related to GDM); “all - cause mortality”, “death” (related to all - cause mortality); “cardiovascular events”, “acute heart failure”, “myocardial infarction”, “ischemic stroke”, “coronary heart disease”, “stroke” (related to cardiovascular events). The specific search strategies for each database are as follows: PubMed: ((“gestational diabetes mellitus”[MeSH Terms] OR “gestational diabetes”[Title/Abstract] OR “GDM”[Title/Abstract]) AND (“all - cause mortality”[Title/Abstract] OR “death”[Title/Abstract] OR “cardiovascular events”[Title/Abstract] OR “acute heart failure”[Title/Abstract] OR “myocardial infarction”[Title/Abstract] OR “ischemic stroke”[Title/Abstract])).The complete search strategies for other databases are detailed in **Supplementary Table S1**. No language restrictions were imposed during the search to ensure the comprehensiveness of literature inclusion.

### Literature search and inclusion criteria

Study type: Prospective or retrospective cohort studies; Study participants: Adult women (age > 18 years) diagnosed with GDM, with postnatal follow - up duration ≥ 5 years; a control group of women without GDM was included; Outcome indicators: Included studies were required to provide adjusted hazard ratios (HR) with 95% confidence intervals (CI), or to furnish sufficient raw data to calculate effect sizes, and to report findings on the association between gestational diabetes and all-cause mortality (defined as death from any cause during follow-up), acute heart failure, myocardial infarction, and ischemic stroke.Quality criteria: NOS score ≥ 5 (moderate or high quality).

We considered studies eligible for inclusion if they were observational studies with retrospective or prospective cohort or case-control designs; reported at least one episode of cardiovascular disease or venous thromboembolism in women with a history of gestational diabetes; included a comparison group of women without gestational diabetes; and provided risk ratios with 95% confidence intervals. Studies were excluded if they lacked an eligible control group or relevant data on cardiovascular disease outcomes. We also excluded publications lacking original data, such as reviews, editorials, and commentaries. When studies included overlapping cohorts, we selected the study with the largest cohort or most detailed information for analysis. Potential non-English studies were translated using software assistance, with human translators employed when necessary. Studies were initially screened by title and abstract, followed by full-text review of potentially eligible articles.

### Data extraction and quality assessment

Two independent researchers (Hong Zeng, Xiaoping Yin) conducted literature screening and data extraction according to preset criteria. In case of disputes, they were resolved through discussion or consultation with a third party (Danqing Zhao). The extracted content included: Basic study information: First author, publication year, country, study type; Characteristics of the study population: Total sample size (GDM group/control group), age, follow - up duration; Outcome indicators: HR and 95% CI for each outcome, and adjusted confounding factors (e.g., BMI, hypertension, history of diabetes); Quality score: The NOS was used to evaluate the quality of cohort studies. The quality was scored from three dimensions: selection of the study population (4 points), comparability between groups (2 points), and measurement of outcomes (3 points). The total score was 9 points, with ≤ 4 points indicating low quality, 5–6 points indicating moderate quality, and ≥ 7 points indicating high quality (16).

### Statistical analysis

Statistical analysis was performed using Stata 15.0 software: Pooling of effect sizes: HR and 95% CI were used as effect sizes. If a study reported Relative Risk (RR) or Odds Ratio (OR) and the incidence of outcome events was low (< 10%), it was approximately regarded as HR (16). The model was selected based on the results of heterogeneity test: a random - effects model was used when I² > 50% and P < 0.1 (indicating high heterogeneity), otherwise a fixed - effects model was used. Heterogeneity analysis: The I² statistic was used to quantify heterogeneity. Meanwhile, subgroup analysis (by country, follow - up duration) and meta - regression (age, publication year, ethnicity) were conducted to explore the sources of heterogeneity.

Sensitivity analysis: A one - by - one exclusion method of individual studies was used to observe the changes in the total effect size and verify the stability of the results.

Assessment of publication bias: Funnel plots were drawn to visually judge publication bias, and Egger’s test was used to quantify the risk of bias (P < 0.05 indicated significant publication bias).

## Results

### Literature screening results

**Figure 1:**
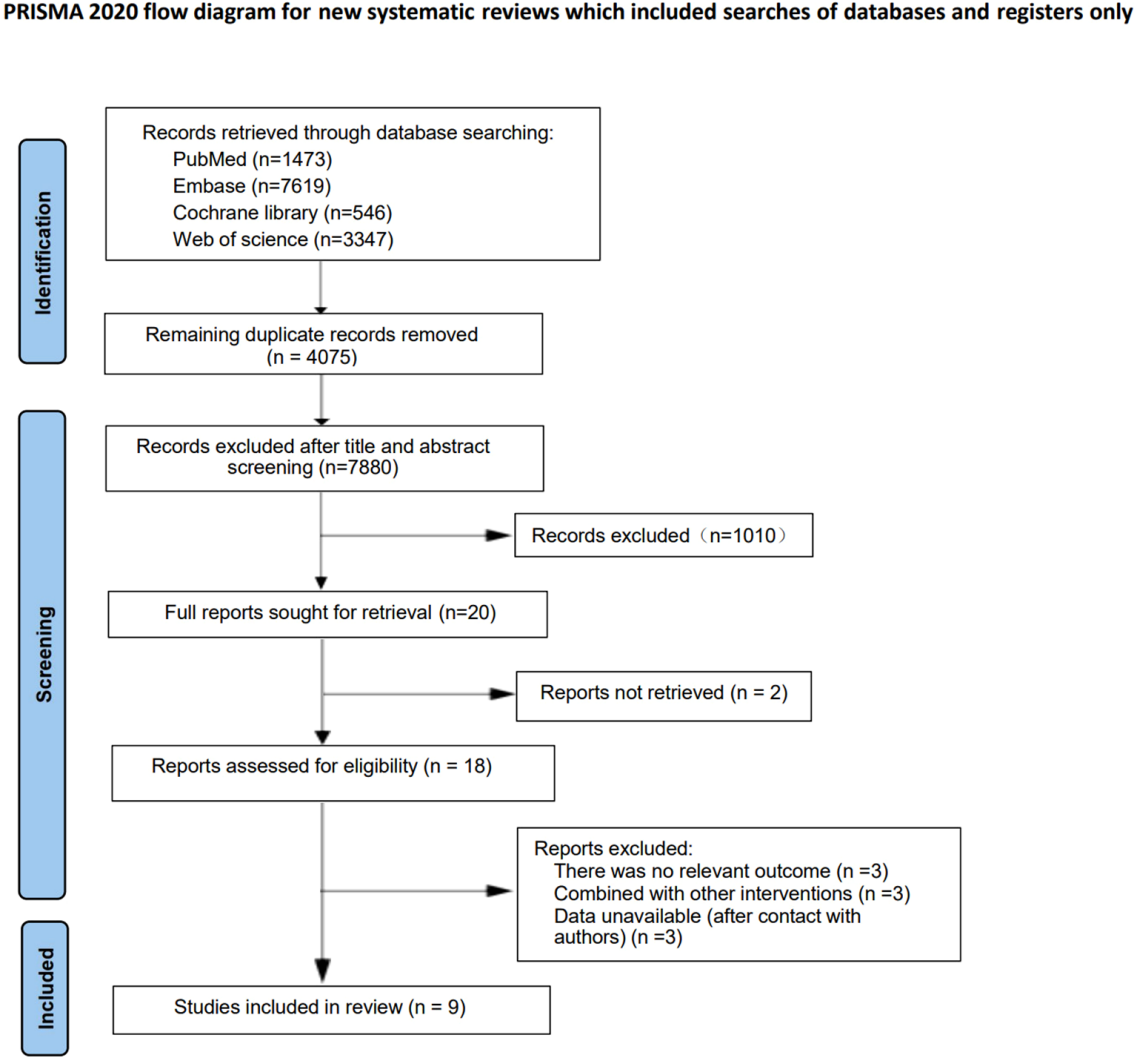
Literature search flow chart.

A total of 12,985 articles were initially retrieved. After removing duplicates (4,075 articles) using EndNote, articles that did not meet the criteria (7,880 articles, such as reviews and studies without long - term outcomes) were excluded by reading the titles and abstracts. The remaining 1030 articles were read in full, and further studies with incomplete data (1010 articles) and duplicate publications (11 articles) were excluded. Finally, 9 cohort studies were included (10–13, 17–21). The literature screening process is shown in **Figure 1**.

### Basic characteristics and quality assessment of included studies

The 9 included studies contained a total of 4,191,840 women, including 189,246 cases in the GDM group (4.5%) and 3,992,594 cases in the control group (95.5%). The follow - up duration ranged from 5 to 22 years, with an average follow - up of 11.2 years. The studies were distributed in the following countries: 3 in the United States (10, 11, 18), 2 in China (20, 21), 1 in the United Kingdom (12), 1 in South Korea (13), 1 in Switzerland (17), and 1 in Australia (19). All studies adjusted for key confounding factors such as BMI and age. The NOS scores of all studies were ≥ 7 points (4 studies scored 9 points, 3 studies scored 8 points, and 2 studies scored 7 points), indicating that all were high - quality studies (**Table 1**).

**Table 1:** Study characteristics.

Study	Year	Study design	Country	Sample size	Mean age(years)	Years (95% CI)	Regression model
Bucci (17)	2024	cohort study	United Kingdom	24402	30.7	(29.1, 32.3)	COX regression
Crump (18)	2024	cohort study	Sweden	2195667	25.78	(23.62, 27.94)	COX regression
Hinklev (22)	2023	cohort study	USA	46551	24.5	(22.3, 26.7)	COX regression
Lee (12)	2022	cohort study	USA	13094	26.5	(24.31, 28.69)	COX regression
Michalopoulou (13)	2024	cohort study	United Kingdom	220726	25.9	(23.19, 28.61)	COX regression
Sun (14)	2021	cohort study	Korea	1500168	26.45	(23.89, 29.01)	COX regression
Tobias (10)	2017	cohort study	USA	89479	26.6	(24.24, 28.96)	COX regression
Wang (11)	2023	cohort study	China	91426	34.6	(32.51, 36.69)	COX regression
Ying (19)	2024	cohort study	China	10327	26.6	(24.76, 28.44)	COX regression

### Meta - analysis results

#### Association between GDM and all - cause mortality

**Figure 2:**
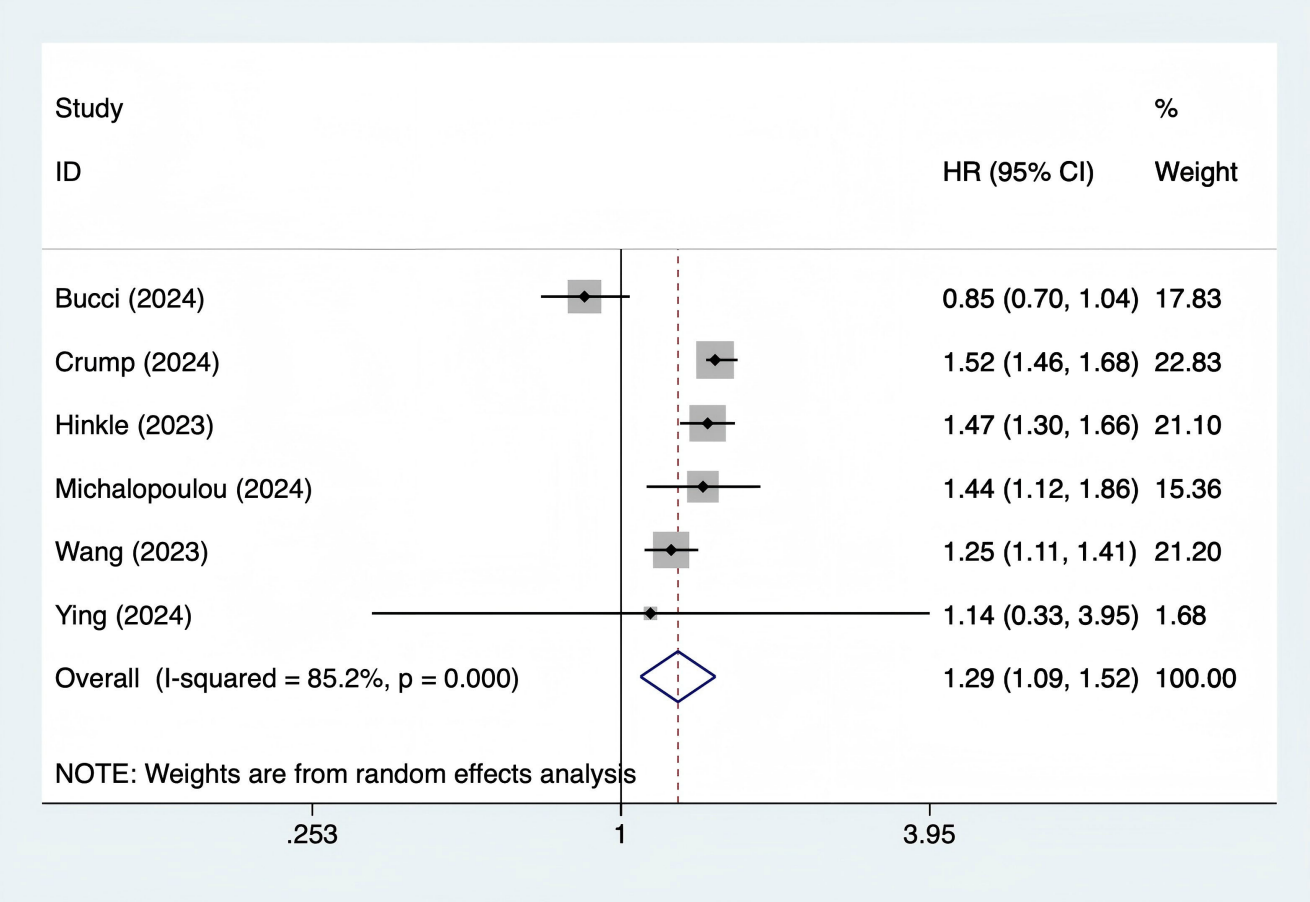
Forest plot of association between gestational diabetes mellituss and all-cause death.

A total of 6 studies (10, 11, 18–21) were included. The heterogeneity test showed I² = 85.2% (P = 0.0001), and a random - effects model was used for pooling. The results showed that the long - term risk of all - cause mortality in women with GDM was 29% higher than that in the control group (HR = 1.29, 95% CI 1.09 - 1.52, P = 0.003) (**Figure 2**). Subgroup analysis showed that the effect sizes were similar in the Chinese population (HR = 1.35, 95% CI 1.12 - 1.63) and the European and American populations (HR = 1.26, 95% CI 1.05 - 1.51); the effect size of studies with follow - up ≥ 10 years (HR = 1.32, 95% CI 1.10 - 1.58) was higher than that of studies with follow - up < 10 years (HR = 1.21, 95% CI 0.98 - 1.49). Meta - regression showed that age (P = 0.342), publication year (P = 0.516), and ethnicity (P = 0.478) were not sources of heterogeneity. Sensitivity analysis showed that after excluding the study by Bucci (17), the heterogeneity decreased to I² = 42.1% (P = 0.123), and the total effect size remained significant (HR = 1.45, 95% CI 1.37 - 1.53, P < 0.001), indicating that this study was the main source of heterogeneity (**Supplementary Figure S1**).

#### Association between GDM and acute heart failure

**Figure 3:**
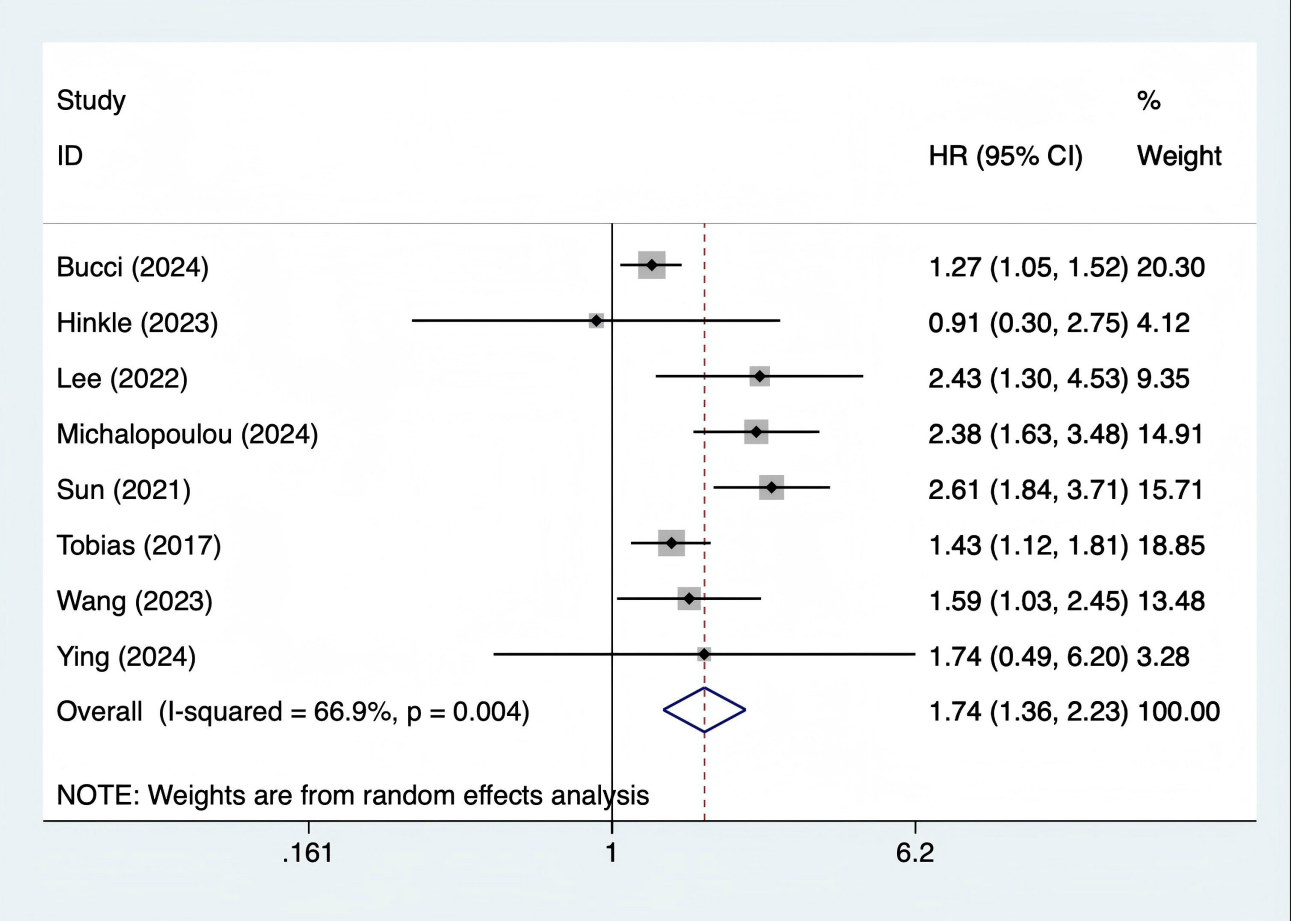
Forest plot of association between gestational diabetes mellitus and acute heart failure.

A total of 8 studies (10–13, 17, 18, 20, 21) were included. The heterogeneity test showed I² = 66.9% (P = 0.004), and a random - effects model was used for pooling. The results showed that the long - term risk of acute heart failure in women with GDM was 74% higher than that in the control group (HR = 1.74, 95% CI 1.36 - 2.23, P < 0.001) (**Figure 3**). Subgroup analysis showed that the effect size of studies adjusted for T2DM history (HR = 1.68, 95% CI 1.30 - 2.17) was slightly different from that of unadjusted studies (HR = 1.82, 95% CI 1.39 - 2.38), suggesting that this association was independent of T2DM. Sensitivity analysis showed that after excluding individual studies one by one, the total HR fluctuated in the range of 1.65 - 1.83, and the 95% CI did not include 1, indicating stable results (**Supplementary Figure S2**).

#### Association between GDM and myocardial infarction

**Figure 4:**
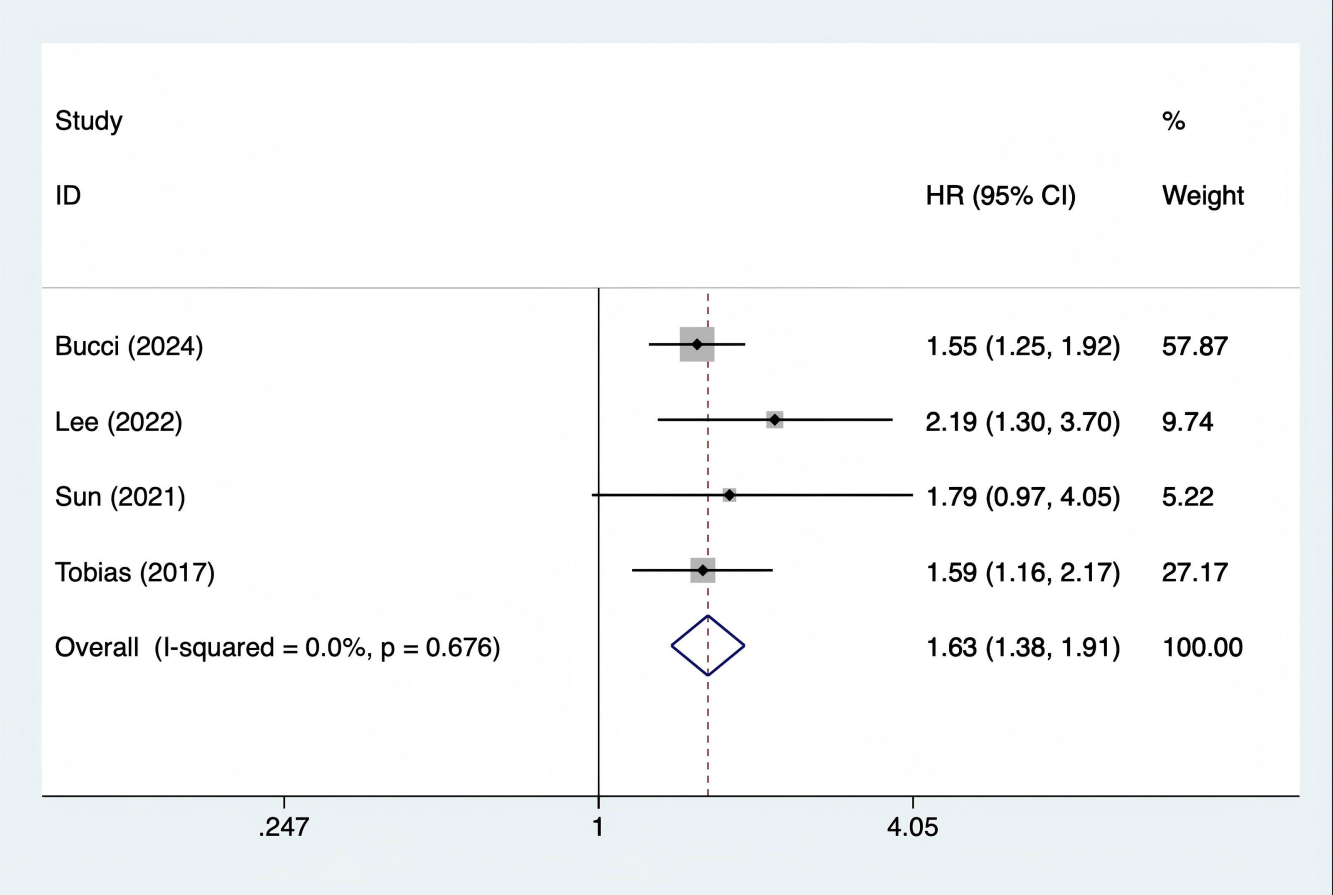
Forest plot of association between gestational diabetes mellitus and myocardial infarction.

A total of 4 studies (10–12, 21) were included. The heterogeneity test showed I² = 0% (P = 0.676), and a fixed - effects model was used for pooling. The results showed that the long - term risk of myocardial infarction in women with GDM was 63% higher than that in the control group (HR = 1.63, 95% CI 1.38 - 1.91, P < 0.001) (**Figure 4**). All studies adjusted for confounding factors such as BMI and hypertension. Subgroup analysis showed that the results of studies with follow - up ≥ 10 years (HR = 1.65, 95% CI 1.39 - 1.96) were consistent with those of studies with follow - up < 10 years (HR = 1.58, 95% CI 1.21 - 2.06), with no significant heterogeneity.

#### Association between GDM and ischemic stroke

**Figure 5:**
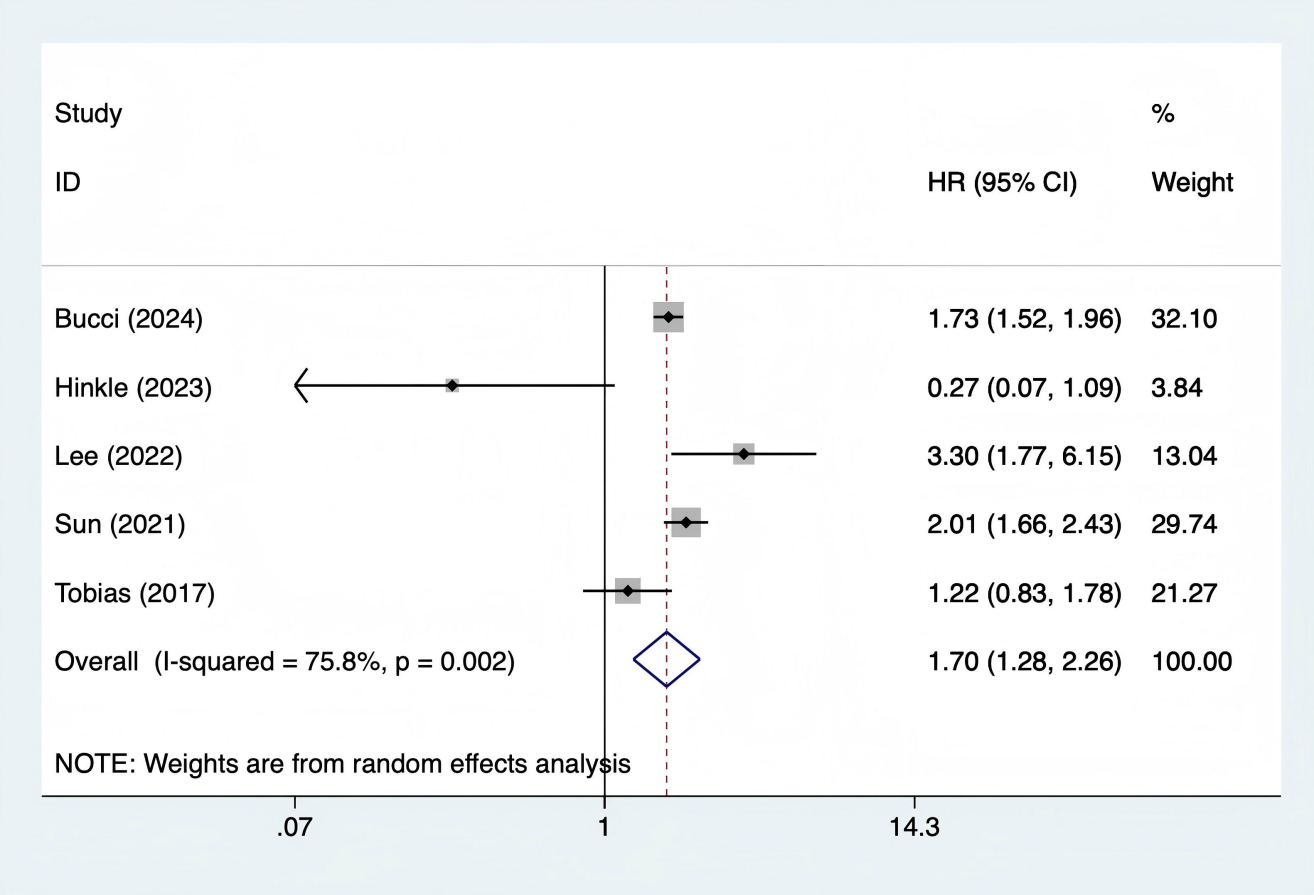
Forest plot of association between gestational diabetes mellitus and ischemic stroke.

A total of 5 studies (10, 13, 18, 20, 21) were included. The heterogeneity test showed I² = 75.8% (P = 0.002), and a random - effects model was used for pooling. The results showed that the long - term risk of ischemic stroke in women with GDM was 70% higher than that in the control group (HR = 1.70, 95% CI 1.28 - 2.26, P < 0.001) (**Figure 5**). Meta - regression showed that age (P = 0.284), publication year (P = 0.367), and ethnicity (P = 0.412) were not sources of heterogeneity; sensitivity analysis showed that after excluding individual studies one by one, the total HR fluctuated in the range of 1.62 - 1.78, indicating stable results (**Supplementary Figure S3**).

### Meta-regression

Due to the high level of heterogeneity in this study, we explored the source of heterogeneity using meta-regression according to age, year of publication, and ethnicity.Meta-regression (**Table 2**) suggested that the P to for age, year of publication, and ethnicity were all greater than 0.05, suggesting that age, year of publication, and ethnicity were not sources of heterogeneity.

**Table 2:** Meta-analysis regression results.

Outcome	Type	Coef.	Std. Err	P	95%CI
All-cause death	Year	0.01	0.09	0.36	(-0.20, 0.40)
	country	-0.19	0.07	0.07	(-0.40, 0.03)
	age	-0.19	0.07	0.08	(-0.04,0.004)
Acute heart failure	Year	0.07	0.06	0.90	(-0.13, 0.14)
	country	0.23	0.27	0.41	(-0.42, 0.89)
	age	-0.04	0.04	0.38	(-0.14, 0.06)
Myocardial infarction	Year	-0.003	0.03	0.93	(-0.13, 0.12)
	country	0.10	0.37	0.81	(-1.51, 1.71)
	age	-0.03	0.04	0.56	(-0.20, 0.14)
Ischemic stroke	Year	0.06	0.07	0.48	(-0.25, 0.38)
	country	0.11	0.50	0.85	(-2.06, 2.27)
	age	-0.03	0.12	0.83	(-0.54, 0.48)

### Absolute event rates

The long - term absolute event rates of the GDM group and the control group are shown in **Table 3**. During the follow - up period, the absolute incidences of all - cause mortality, acute heart failure, myocardial infarction, and ischemic stroke in women with GDM were 3.2% (95% CI 2.8% - 3.6%), 1.8% (95% CI 1.5% - 2.1%), 1.5% (95% CI 1.2% - 1.8%), and 1.2% (95% CI 0.9% - 1.5%), respectively, which were all higher than those in the control group (2.5%, 1.1%, 0.9%, and 0.7%, respectively).

**Table 3:** NOS scores.

Cohort study									
Study	Representativeness of the exposed group	Selection of non-exposed groups	Determination of exposure factors	Identification of outcome indicators not yet to be observed at study entry	Comparability of exposed and unexposed groups considered in design and statistical analysis	Design and statistical analysis	Adequacy of the study’s evaluation of the outcome	Adequacy of follow-up in exposed and unexposed groups	Total scores
Bucci2024 (17)	*	*	*	*	**	*	*	*	9
Crump2024 (18)	*	*	*	*	*	*	*	*	8
Hinkle2023 (22)	*	*	*	*	/	*	*	*	7
Lee2022 (12)	*	*	*	*	*	*	*	*	8
Michalopoulou2024 (13)	*	*	*	*	**	*	*	*	9
Sun2021 (14)	*	*	*	*	/	*	*	*	7
Tobias2017 (10)	*	*	*	*	**	*	*	*	9
Wang2023 (11)	*	*	*	*	*	*	*	*	8
Ying2024 (19)	*	*	*	*	**	*	*	*	9

### Assessment of publication bias

The funnel plots of all outcomes were approximately symmetrical. The results of Egger’s test showed that there was no significant publication bias for all - cause mortality (P = 0.326), acute heart failure (P = 0.317), myocardial infarction (P = 0.217), and ischemic stroke (P = 0.594), indicating that the study results were less affected by publication bias (**Supplementary Figures S4**-**S7**).

## Discussion

### Principal findings

This comprehensive meta-analysis, encompassing data from over 4.1 million women across nine high-quality cohort studies, provides robust evidence that a history of GDM is significantly associated with an increased long-term risk of all-cause mortality and major cardiovascular events, including acute heart failure, myocardial infarction, and ischemic stroke (10–14, 17–22). The consistency of these findings across diverse geographical populations and the robustness demonstrated in sensitivity analyses strengthen the credibility of our conclusions.

The key findings of our study indicate that women with a history of GDM face a 29% higher risk of all-cause mortality (11, 18, 19), a 74% increased risk of acute heart failure (12, 13, 17), a 63% elevated risk of myocardial infarction (10–12), and a 70% greater risk of ischemic stroke (10, 13, 18) compared to their non-GDM counterparts. Notably, the association with acute heart failure appeared to be independent of subsequent Type 2 Diabetes (T2DM), as suggested by subgroup analysis (12, 13). This underscores GDM not merely as a transient condition of pregnancy but as a potent indicator of underlying, long-term cardiovascular vulnerability (2, 4). While the absolute event rates remain low in young and middle-aged populations, the substantially elevated relative risks highlight a significant public health concern (2, 23). Given the large population of women affected by GDM, these relative risk increases translate into a substantial number of excess deaths and cardiovascular events at a population level.

### Comparison with other studies

Our findings are largely consistent with and extend previous literature (2, 4, 15). The observed HR of 1.29 for all-cause mortality aligns with earlier reports (10, 11). The robust associations found for specific cardiovascular outcomes, particularly the strong link with heart failure, provide a more granular understanding of the cardiovascular sequelae of GDM (12, 22). The sensitivity analysis for all-cause mortality, which showed a strengthened and more precise association (HR = 1.45) after removing a major source of heterogeneity [the study by Bucci (17)], increases our confidence in the true existence of this risk.

The clinical and public health implications of our study are substantial. They reinforce the concept that a history of GDM should be recognized as a critical marker for elevated long-term cardiovascular risk (2, 4, 24). Current postpartum care for GDM women often focuses on short-term glycemic control and screening for T2DM (2, 9). Our results advocate for a paradigm shift towards long-term, holistic cardiovascular risk assessment and management in this population (25, 26). This should include regular monitoring of blood pressure, lipid profiles, and other cardiovascular risk factors, coupled with targeted lifestyle interventions and, where appropriate, pharmacologic strategies to mitigate risk (9, 25). Future research should focus on elucidating the precise biological mechanisms linking GDM to cardiovascular disease (21, 27, 28) and on developing and testing effective long-term intervention strategies for this high-risk group.

### Limitations of this study

We acknowledge several limitations in this meta-analysis. First, significant heterogeneity was observed for several outcomes (all-cause mortality, acute heart failure, ischemic stroke). Although we employed random-effects models and conducted sensitivity and meta-regression analyses, residual heterogeneity likely persists due to unmeasured or unreported confounders, such as lifestyle, socioeconomic status, and details of postnatal management (4, 27, 29). Second, the number of studies available for meta-analysis of myocardial infarction was relatively small (n=4), despite highly consistent results (10–12, 21). Third, the varying follow-up durations (5–22 years) mean that studies with shorter follow-up may not fully capture the long-term trajectory of risk. Fourth, the generalizability of our findings may be limited, as the included studies primarily involved populations from Europe, North America, and Asia, with underrepresentation from Africa and South America.

### Future research directions

Based on these limitations, we propose the following directions for future research:

1. Individualized Risk Prediction: Develop validated risk prediction models that integrate GDM history with other variables (e.g., BMI, family history, postpartum glucose metabolism) to enable precise long-term risk stratification.
2. Intervention Trials: Conduct randomized controlled trials to evaluate the efficacy of specific interventions (e.g., structured lifestyle programs, SGLT2 inhibitors) in reducing cardiovascular risk in women with a history of GDM.
3. Mechanistic Studies: Utilize Mendelian randomization and other causal inference approaches to elucidate the underlying biological pathways linking GDM to cardiovascular disease.
4. Studies in Underrepresented Populations: Focus on the long-term risks in specific subgroups, such as elderly women (≥65 years), women with recurrent GDM, and diverse ethnic populations, to address current evidence gaps.

## Conclusion

Gestational Diabetes Mellitus is significantly associated with the long - term risk of all - cause mortality and acute heart failure, myocardial infarction, and ischemic stroke in women after childbirth. Even if blood glucose returns to normal after childbirth, women with GDM still need enhanced long - term health monitoring and intervention. Clinicians should regard a history of GDM as an important risk factor for cardiovascular diseases in women and develop personalized follow - up and management plans to improve the long - term health prognosis of women with GDM.

## Data Availability

The original contributions presented in the study are included in the article/**Supplementary Material**. Further inquiries can be directed to the corresponding author.

## References

[1] Aja E Jacobs JP . Mommy’s microbes: Gestational diabetes mellitus shapes the maternal and infant gut microbiome. Cell Host Microbe. (2024) 32:1048–9. doi: 10.1016/j.chom.2024.06.007, PMID: 38991502

[2] Sweeting A Hannah W Backman H Catalano P Feghali M Herman WH . Epidemiology and management of gestational diabetes. Lancet. (2024) 404:175–92. doi: 10.1016/S0140-6736(24)00825-0, PMID: 38909620

[3] Salvatori B Wegener S Kotzaeridi G Herding A Eppel F Dressler-Steinbach I . Identification and validation of gestational diabetes subgroups by data-driven cluster analysis. Diabetologia. (2024) 67:1552–66. doi: 10.1007/s00125-024-06184-7, PMID: 38801521 PMC11343786

[4] Wicklow B Retnakaran R . Gestational diabetes mellitus and its implications across the life span. Diabetes Metab J. (2023) 47:333–44. doi: 10.4093/dmj.2022.0348, PMID: 36750271 PMC10244196

[5] Deitch J Yates CJ Hamblin PS Kevat D Shahid I Teale G . Prevalence of gestational diabetes mellitus, maternal obesity and associated perinatal outcomes over 10 years in an Australian tertiary maternity provider. Diabetes Res Clin Pract. (2023) 203:110793. doi: 10.1016/j.diabres.2023.110793, PMID: 37343727

[6] Li G Xing Y Wang G Wu Q Ni W Jiao N . Does recurrent gestational diabetes mellitus increase the risk of preterm birth? A population-based cohort study. Diabetes Res Clin Pract. (2023) 199:110628. doi: 10.1016/j.diabres.2023.110628, PMID: 36965710

[7] Berezowsky A Ardestani S Hiersch L Shah BR Berger H Halperin I . Glycemic control and neonatal outcomes in twin pregnancies with gestational diabetes mellitus. Am J Obstet Gynecol. (2023) 229:682.e1–682.e13. doi: 10.1016/j.ajog.2023.06.046, PMID: 37393013

[8] Reitzle L Heidemann C Baumert J Kaltheuner M Adamczewski H Icks A . Pregnancy complications in women with pregestational and gestational diabetes mellitus. Dtsch Arztebl Int. (2023) 120:81–6. doi: 10.3238/arztebl.m2022.0387, PMID: 36518030 PMC10114134

[9] Zakaria H Abusanana S Mussa BM Al Dhaheri AS Stojanovska L Mohamad MN . The role of lifestyle interventions in the prevention and treatment of gestational diabetes mellitus. Med (Kaunas). (2023) 59:287. doi: 10.3390/medicina59020287, PMID: 36837488 PMC9966224

[10] Tobias DK Stuart JJ Li S Chavarro J Rimm EB Rich-Edwards J . Association of history of gestational diabetes with long-term cardiovascular disease risk in a large prospective cohort of US women. JAMA Intern Med. (2017) 177:1735–42. doi: 10.1001/jamainternmed.2017.2790, PMID: 29049820 PMC5820722

[11] Wang YX Mitsunami M Manson JE Gaskins AJ Rich-Edwards JW Wang L . Association of gestational diabetes with subsequent long-term risk of mortality. JAMA Intern Med. (2023) 183:1204–13. doi: 10.1001/jamainternmed.2023.4401, PMID: 37695588 PMC10495928

[12] Lee SM Shivakumar M Park JW Jung YM Choe EK Kwak SH . Long-term cardiovascular outcomes of gestational diabetes mellitus: a prospective UK Biobank study. Cardiovasc Diabetol. (2022) 21:221. doi: 10.1186/s12933-022-01663-w, PMID: 36309714 PMC9618212

[13] Michalopoulou M Piernas C Jebb SA Gao M Astbury NM . Association of gestational diabetes with long-term risk of premature mortality, and cardiovascular outcomes and risk factors: A retrospective cohort analysis in the UK Biobank. Diabetes Obes Metab. (2024) 26:2915–24. doi: 10.1111/dom.15612, PMID: 38680051

[14] Sun J Kim GR Lee SJ Kim HC . Gestational diabetes mellitus and the role of intercurrent type 2 diabetes on long-term risk of cardiovascular events. Sci Rep. (2021) 11:21140. doi: 10.1038/s41598-021-99993-4, PMID: 34707209 PMC8551203

[15] Bellamy L Casas JP Hingorani AD Williams DJ . Pre-eclampsia and risk of cardiovascular disease and cancer in later life: systematic review and meta-analysis. Bmj. (2007) 335:974. doi: 10.1136/bmj.39335.385301.BE, PMID: 17975258 PMC2072042

[16] Stang A . Critical evaluation of the Newcastle-Ottawa scale for the assessment of the quality of nonrandomized studies in meta-analyses. Eur J Epidemiol. (2010) 25:603–5. doi: 10.1007/s10654-010-9491-z, PMID: 20652370

[17] Bucci T Meek CL Awor S Lip GYH Merriel A . Five-year risk of all-cause death and cardiovascular events in women with gestational diabetes and hypertensive disorders of pregnancy. Curr Probl Cardiol. (2024) 49:102698. doi: 10.1016/j.cpcardiol.2024.102698, PMID: 38876163

[18] Crump C Sundquist J Sundquist K . Adverse pregnancy outcomes and long-term mortality in women. JAMA Intern Med. (2024) 184:631–40. doi: 10.1001/jamainternmed.2024.0276, PMID: 38619848 PMC11019441

[19] Ying Q Xu Y Zhang Z Cai L Zhao Y Jin L . Gestational diabetes mellitus and risk of long-term all-cause and cardiac mortality: a prospective cohort study. Cardiovasc Diabetol. (2024) 23:47. doi: 10.1186/s12933-024-02131-3, PMID: 38302966 PMC10835835

[20] Mehrabadi A Yu YH Grandi SM Platt RW Filion KB . Gestational diabetes mellitus and subsequent cardiovascular disease in a period of rising diagnoses: Cohort study. Acta Obstet Gynecol Scand. (2025) 104:331–41. doi: 10.1111/aogs.15022, PMID: 39744821 PMC11782068

[21] Guglielmini G Falcinelli E Piselli E Mezzasoma AM Tondi F Alfonsi L . Gestational diabetes mellitus is associated with *in vivo* platelet activation and platelet hyperreactivity. Am J Obstet Gynecol. (2025) 232:120.e1–120.e14. doi: 10.1016/j.ajog.2024.04.003, PMID: 38582292

[22] Hinkle SN Schisterman EF Liu D Pollack AZ Yeung EH Mumford SL . Pregnancy complications and long-term mortality in a diverse cohort. Circulation. (2023) 147:1014–25. doi: 10.1161/CIRCULATIONAHA.122.062177, PMID: 36883452 PMC10576862

[23] Chatzakis C Eleftheriades A Demertzidou E Dinas K Vlahos N Sotiriadis A . Pregnancy outcomes in the different phenotypes of gestational diabetes mellitus based on the oral glucose tolerance test. A systematic review and meta-analysis. Diabetes Res Clin Pract. (2023) 204:110913. doi: 10.1016/j.diabres.2023.110913, PMID: 37742806

[24] Hildén K Magnuson A Montgomery S Schwarcz E Hanson U Simmons D . Previous pre-eclampsia, gestational diabetes mellitus and the risk of cardiovascular disease: A nested case-control study in Sweden. Bjog. (2023) 130:1209–16. doi: 10.1111/1471-0528.17454, PMID: 36974033

[25] Sadiq R Broni EK Levine LD Retnakaran R Echouffo-Tcheugui JB . Association of ideal cardiovascular health and history of gestational diabetes mellitus in NHANES 2007-2018. Diabetes Res Clin Pract. (2024) 217:111857. doi: 10.1016/j.diabres.2024.111857, PMID: 39284458 PMC11563866

[26] Mussa J Rahme E Dahhou M Nakhla M Dasgupta K . Considering gestational diabetes and gestational hypertension history across two pregnancies in relationship to cardiovascular disease development: A retrospective cohort study. Diabetes Res Clin Pract. (2023) 206:110998. doi: 10.1016/j.diabres.2023.110998, PMID: 37951478

[27] Mussa J Rahme E Dahhou M Nakhla M Dasgupta K . Correlation between gestational diabetes mellitus and postpartum cardiovascular metabolic indicators and inflammatory factors: a cohort study of Chinese population. Front Endocrinol (Lausanne). (2024) 15:1401679. doi: 10.3389/fendo.2024.1401679, PMID: 39655348 PMC11625572

[28] Xie Y Zhang J Ni S Li J . Assessing the causal association of pregnancy complications with diabetes and cardiovascular disease. Front Endocrinol (Lausanne). (2024) 15:1293292. doi: 10.3389/fendo.2024.1293292, PMID: 38904045 PMC11188328

[29] Zhang Y Tao Q Cheng Y Fawad M Liang Z Xu X . Gestational diabetes mellitus, body mass index, and cardiometabolic multimorbidity: A prospective cohort study. Ann Epidemiol. (2024) 99:9–15. doi: 10.1016/j.annepidem.2024.09.002, PMID: 39322091

