# Supplementary material for: Long-term risk of all-cause mortality and cardiovascular events in women with gestational diabetes mellitus: a systematic review and meta-analysis
Source: Front Endocrinol (Lausanne). 2026 Feb 25;17:1646691. doi: 10.3389/fendo.2026.1646691 (PMC12975557; doi:10.3389/fendo.2026.1646691)
Supplement: Supplementary file 1 [file DataSheet1.pdf]

Table S1 search strategy

(("Diabetes, Gestational"[Mesh]) OR ((((((Diabetes, Gestational[MeSH Terms]) OR (Diabetes Mellitus, Gestational[Title/Abstract])) OR (Gestational Diabetes Mellitus[Title/Abstract])) OR (Diabetes, Pregnancy-Induced[Title/Abstract])) OR (Diabetes, Pregnancy Induced[Title/Abstract])) OR (Pregnancy-Induced Diabetes[Title/Abstract])) OR (Gestational Diabetes[Title/Abstract]))) AND (("Mortality"[Mesh]) OR ((Mortality[MeSH Terms]) OR (Mortalities[Title/Abstract])) OR (Mortality Rate[Title/Abstract])) OR (Mortality Rates[Title/Abstract])) OR (Rate, Mortality[Title/Abstract])) OR (Death Rate[Title/Abstract])) OR (Death Rates[Title/Abstract])) OR (Rate, Death[Title/Abstract])) OR (Mortality, Differential[Title/Abstract])) OR (Differential Mortality[Title/Abstract])) OR (Differential Mortalities[Title/Abstract])) OR (Mortality, Excess[Title/Abstract])) OR (Excess Mortality[Title/Abstract])) OR (Excess Mortalities[Title/Abstract])) OR (Mortality Determinants[Title/Abstract])) OR (Determinants, Mortality[Title/Abstract])) OR (Determinant, Mortality[Title/Abstract])) OR (Mortality Determinant[Title/Abstract])) OR (Case Fatality Rate[Title/Abstract])) OR (Case Fatality Rates[Title/Abstract])) OR (Rate, Case Fatality[Title/Abstract])) OR (Rates, Case Fatality[Title/Abstract])) OR (CFR Case Fatality Rate[Title/Abstract])) OR (Decline, Mortality[Title/Abstract])) OR (Mortality Declines[Title/Abstract])) OR (Mortality Decline[Title/Abstract])) OR (Age-Specific Death Rate[Title/Abstract])) OR (Age-Specific Death Rates[Title/Abstract])) OR (Death Rate, Age-Specific[Title/Abstract])) OR (Rate, Age-Specific Death[Title/Abstract])) OR (Age Specific Death Rate[Title/Abstract])) OR (Crude Death Rate[Title/Abstract])) OR (Crude Death Rates[Title/Abstract])) OR (Death Rate, Crude[Title/Abstract])) OR (Rate, Crude Death[Title/Abstract])) OR (Crude Mortality Rate[Title/Abstract])) OR (Crude Mortality Rates[Title/Abstract])) OR (Mortality Rate, Crude[Title/Abstract])) OR (Rate, Crude Mortality[Title/Abstract])) OR (Death[Title/Abstract])) OR (End Of Life[Title/Abstract])) OR (End-Of-Life[Title/Abstract])) OR (Determination of Death[Title/Abstract])) OR (Near-Death Experience[Title/Abstract])) OR (metabolic syndrome[Title/Abstract])) OR (cardiovascular disease [Title/Abstract])))OR "pregnancy in diabetics"[Title/Abstract]] OR("pregnancy"[Title/Abstract]] AND "diabetes"[Title/Abstract]] AND "mellitus"[Title/Abstract]]) OR "pregnancy diabetes mellitus"[Title/Abstract]])) AND ("cardiovascular diseases"[MeSH Terms] OR ("cardiovascular"[Title/Abstract]] AND "diseases"[Title/Abstract]]) OR "cardiovascular diseases"[Title/Abstract]] OR ("venous thromboembolism"[MeSH Terms] OR ("venous"[Title/Abstract]] AND "thromboembolism"[Title/Abstract]]) OR "venous thromboembolism"[Title/Abstract]]) OR ("cardiovascular system"[MeSH Terms] OR ("cardiovascular"[Title/Abstract]] AND "system"[Title/Abstract]]) OR "cardiovascular system"[Title/Abstract]] OR "cardiovascular"[Title/Abstract]] OR "cardiovasculars"[Title/Abstract]]) OR (("coronary vessels"[MeSH Terms] OR ("coronary"[Title/Abstract]] AND "vessels"[Title/Abstract]]) OR "coronary vessels"[Title/Abstract]]OR("coronary"[Title/Abstract]]AND "artery"[Title/Abstract]])

OR "coronary artery"[Title/Abstract]]) AND "disease*"[Title/Abstract]]) OR (("coronaries"[Title/Abstract]] OR "heart"[MeSH Terms] OR "heart"[Title/Abstract]] OR "coronary"[Title/Abstract]]) AND ("heart"[MeSH Terms] OR "heart"[Title/Abstract]]OR"hearts"[Title/Abstract]]OR"hearts"[Title/Abstract]])AND" disease*"[Title/Abstract]])OR("cardiacs"[Title/Abstract]] OR "heart"[MeSH Terms] OR"heart"[Title/Abstract]]OR"cardiac"[Title/Abstract]])OR(("ischaemics"[Title/Abstract]] OR "ischemia"[MeSH Terms]

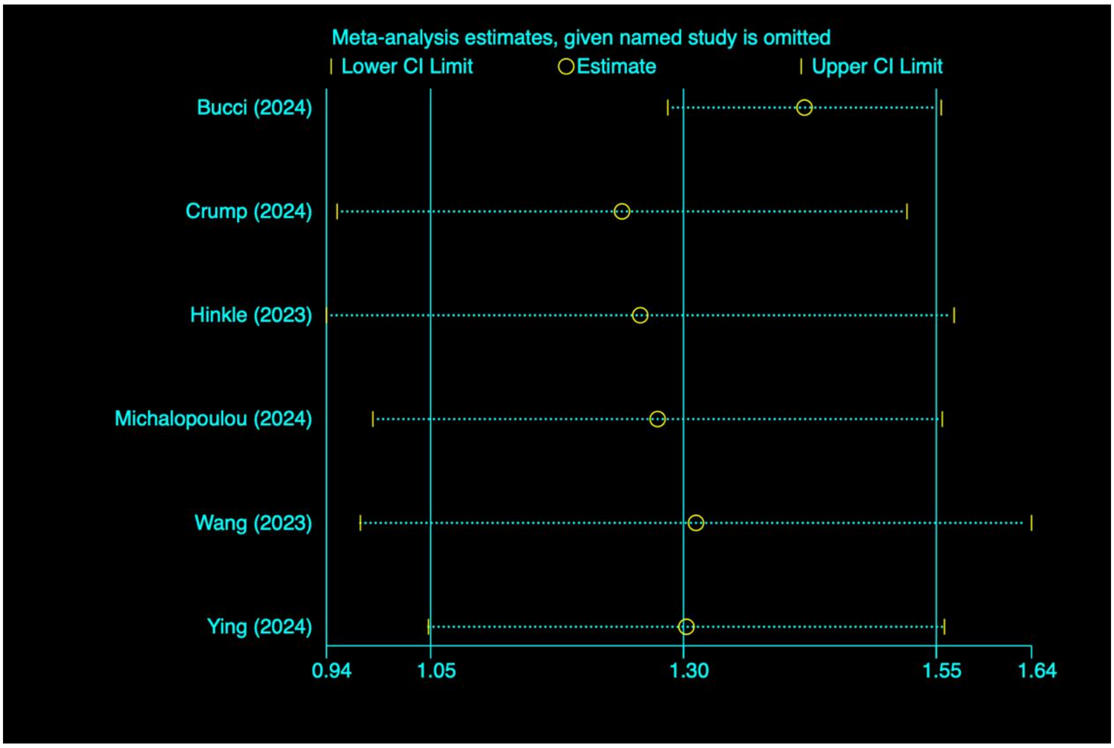

Figure S1 Sensitivity analysis of the Association between gestational diabetes and all-cause death

Sensitivity analysis indicates that the findings of this meta-analysis regarding the association between GDM and all-cause mortality are robust and reliable, and are not unduly influenced by any single study.

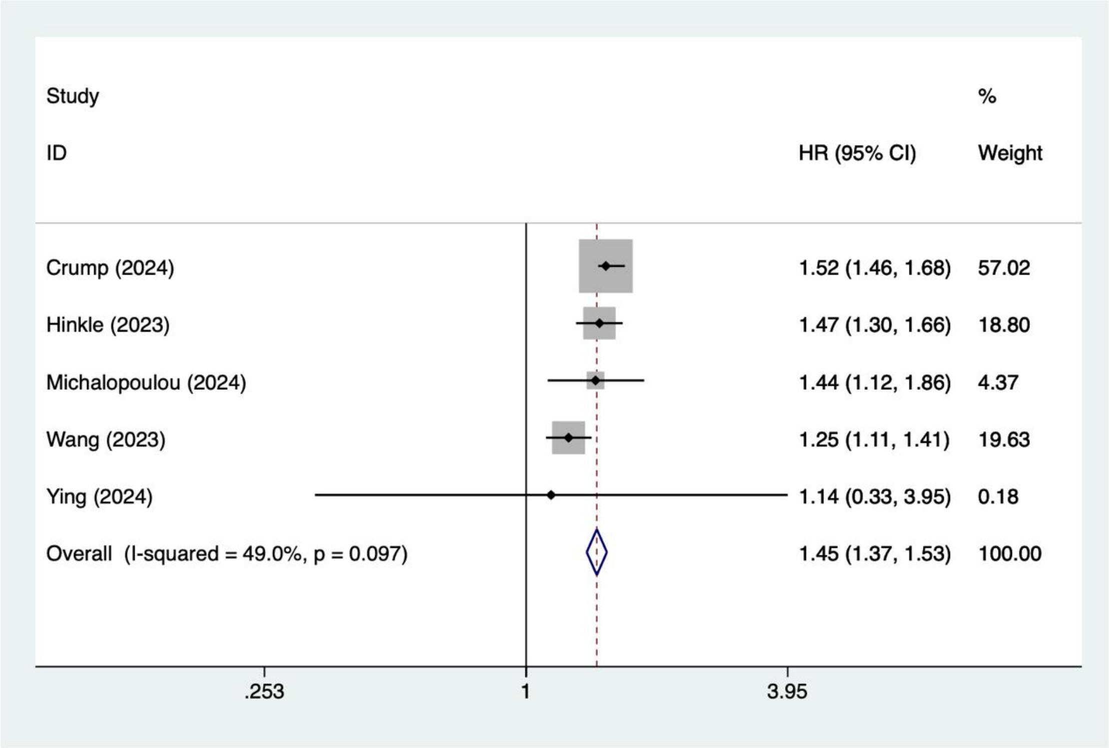

Figure S2 Meta-analysis of Association between gestational diabetes and all-cause death in Bucci2024 was removed

After excluding the Bucci (2024) study, heterogeneity significantly decreased from 85.2% to 49.0%, approaching an acceptable range. The pooled HR increased from 1.29 to 1.45, with a narrower 95% CI (1.37–1.53) and markedly improved statistical precision. Despite study exclusion, the positive association between GDM and all-cause mortality remained robust with strengthened evidence. This suggests the all-cause mortality risk among women with GDM history may be underestimated, underscoring the necessity for long-term follow-up and intervention strategies.

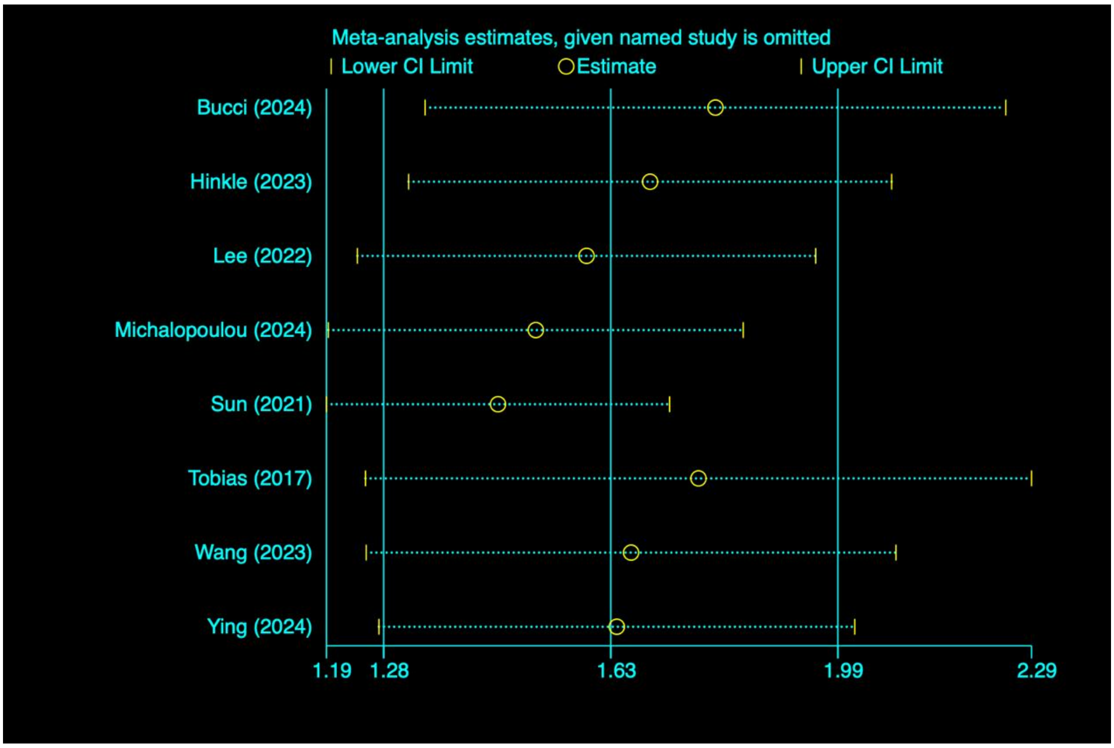

Figure S3 Sensitivity analysis of the Association between gestational diabetes and long-term acute heart failure

Sensitivity analysis confirmed that the association between GDM and acute heart failure (HR ≈ 1.74) was not disproportionately influenced by any single study. Unlike all-cause mortality (where Bucci 2024 was the primary source of heterogeneity), the heterogeneity in acute heart failure likely resulted from multiple studies collectively, with no single dominant study. Regardless of which study was excluded, The risk of acute heart failure in women with GDM remains significantly elevated by approximately 60%-75%, providing robust evidence for clinical intervention. This sensitivity analysis further reinforces the original conclusion: the association between GDM and acute heart failure is independent of type 2 diabetes and represents a significant long-term complication of GDM itself.

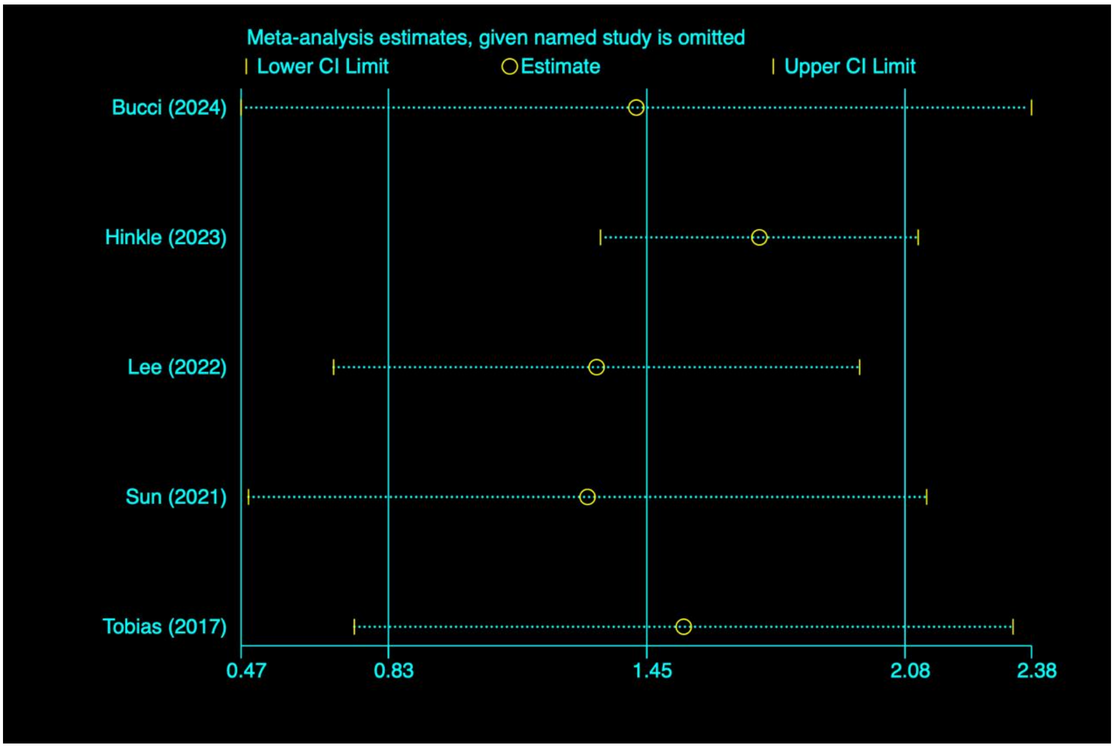

Figure S4 Sensitivity analysis of the Association between gestational diabetes and long-term ischemic stroke

Sensitivity analysis confirmed that the association between GDM and ischemic stroke (HR ≈1.70) remained stable after excluding most individual studies. The Lee (2022) study may have a larger effect size (HR>1.70), and its exclusion resulted in the most significant overall effect reduction, though it still remained around 1.35. Hinkle (2023) may have a smaller effect size, as the overall HR increased after its exclusion, suggesting its effect size might be below average. Regardless of which study was excluded, the risk of ischemic stroke in women with GDM increased by approximately 35%-75%, providing robust evidence supporting long-term follow-up and cerebrovascular disease prevention.

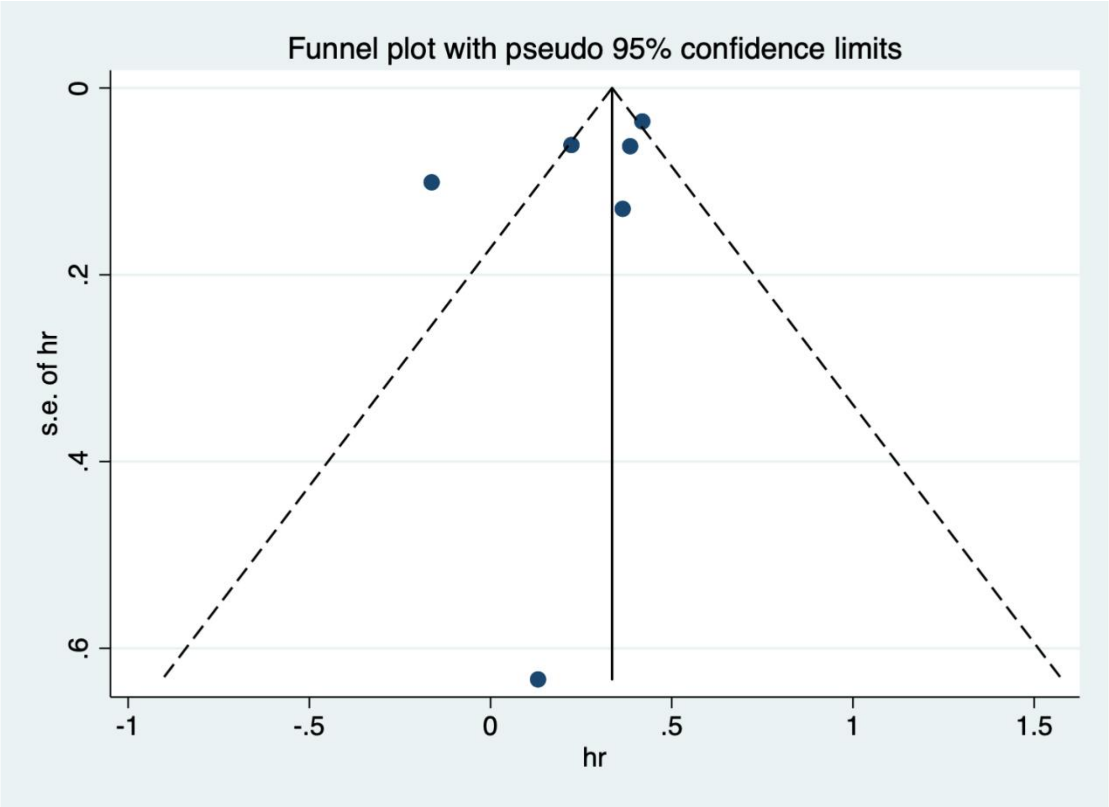

Figure S5 funnel plot of the Association between gestational diabetes and all-cause death

The funnel plot exhibits basic symmetry, with an Egger test P=0.326 > 0.05, indicating that the results of this meta-analysis are minimally affected by publication bias. The distribution of included studies aligns with expectations, and no evidence of systematic exclusion of small-sample negative results was found, suggesting high credibility of the results.

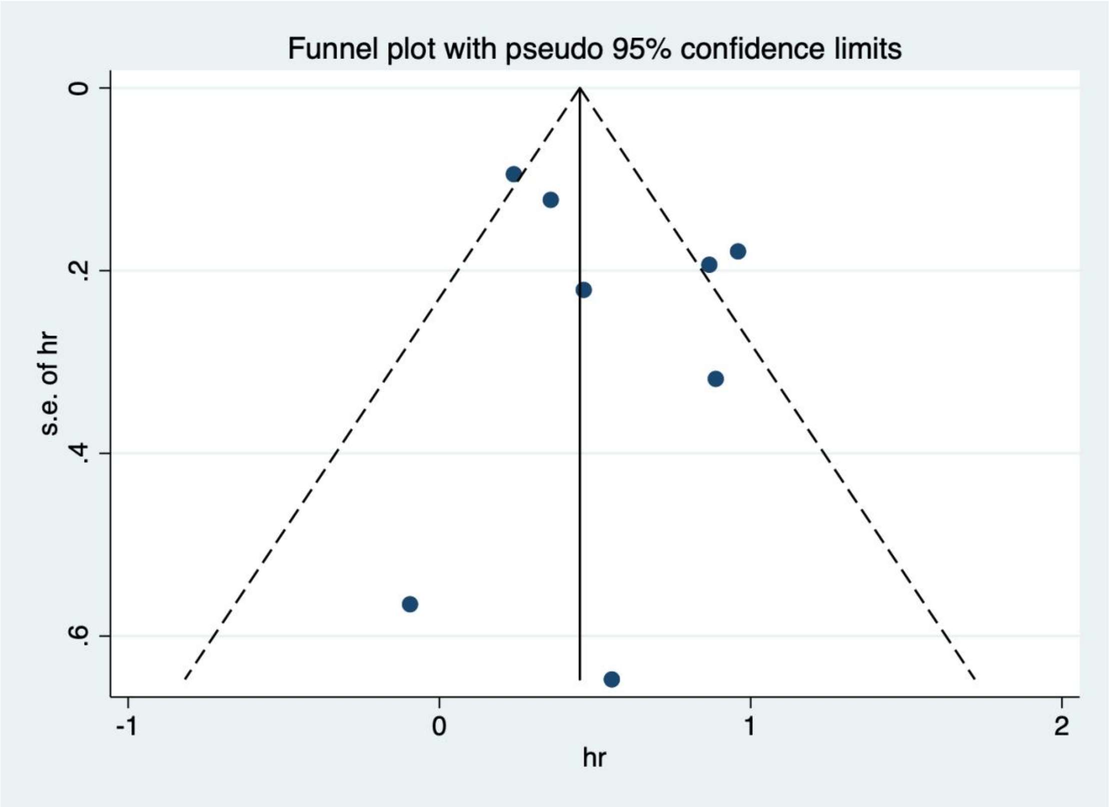

Figure S6 funnel plot of the Association between gestational diabetes and acute heart failure

The funnel plot exhibits basic symmetry, with an Egger test P=0.317 > 0.05, suggesting minimal publication bias in this meta-analysis. The dispersion of study distributions reflects clinical heterogeneity rather than publication bias. Large-sample studies clustered around the pooled effect size, while small-sample studies showed dispersion without systematic bias, supporting the reliability of the association between GDM and acute heart failure. The distribution of acute heart failure studies was more dispersed (I^2=66.9% vs 85.2%), but both conditions exhibited low risk of publication bias.

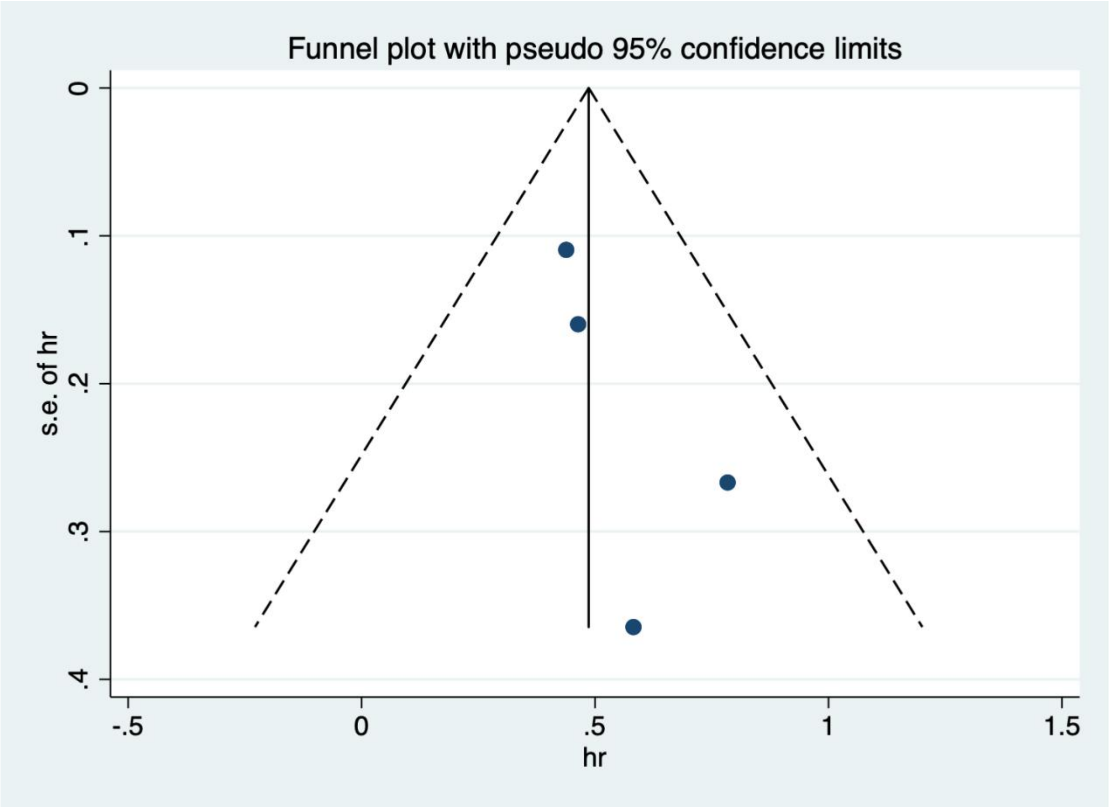

Figure S7 funnel plot of the Association between gestational diabetes and myocardial infarction

Myocardial infarction was the outcome with the lowest heterogeneity in this study (I^2=0%). Results from the four studies showed high consistency, yielding a pooled HR of 1.63 (95% CI 1.38-1.91), which is precise and reliable. Although only four studies were included, limiting the effectiveness of funnel plot analysis, the existing evidence showed no signs of under-reporting negative results from small samples. The association between GDM and myocardial infarction demonstrated the strongest evidence across all outcomes, with no concerns regarding publication bias. This provides the most reliable evidence supporting clinical prevention strategies.

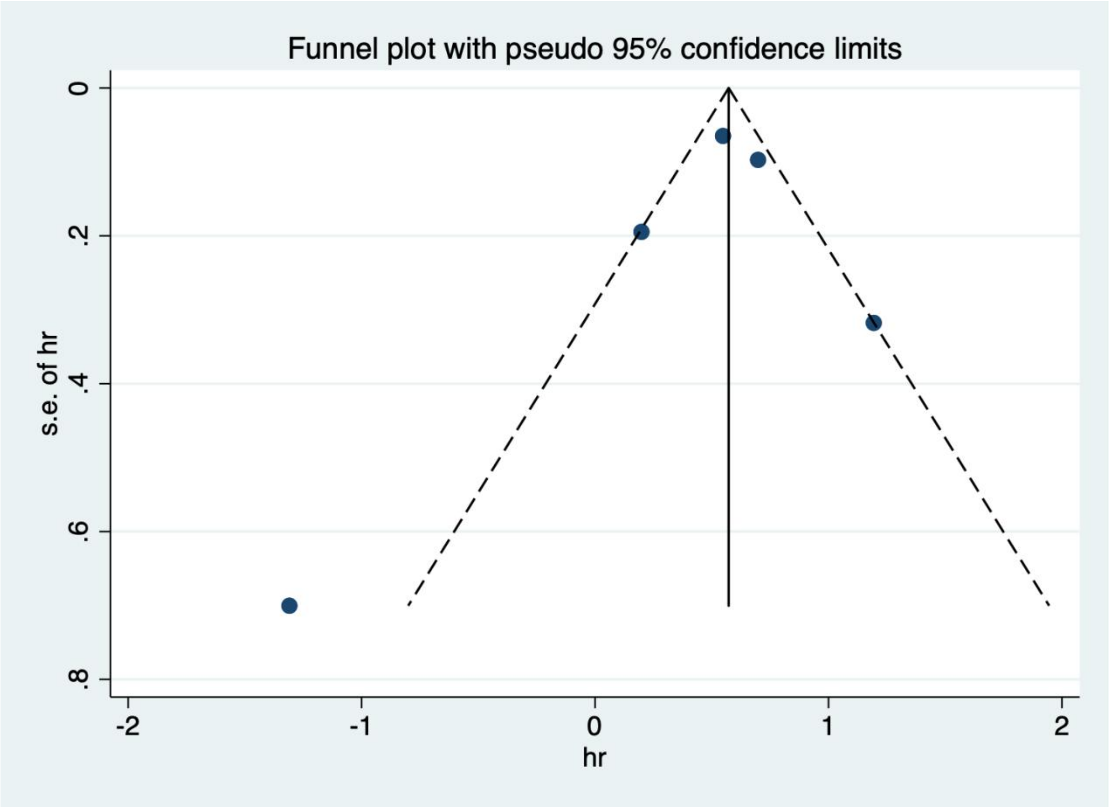

Figure S8 funnel plot of the Association between gestational diabetes and ischemic stroke

I^2 = 75.8% (P = 0.002) for ischemic stroke, with funnel plots visually illustrating this heterogeneity, primarily driven by Hinkle (2023). Excluding Hinkle (2023), the remaining four studies consistently supported an association between GDM and increased ischemic stroke risk, yielding a clinically significant pooled HR of 1.70 (95% CI 1.28–2.26).
